# Metabolomic Profiling of Long‐Term Weight Change: Role of Oxidative Stress and Urate Levels in Weight Gain

**DOI:** 10.1002/oby.21922

**Published:** 2017-07-31

**Authors:** Cristina Menni, Marie Migaud, Gabi Kastenmüller, Tess Pallister, Jonas Zierer, Annette Peters, Robert P. Mohney, Tim D. Spector, Vincenzo Bagnardi, Christian Gieger, Steve C. Moore, Ana M. Valdes

**Affiliations:** ^1^ Department of Twin Research & Genetic Epidemiology King's College London London UK; ^2^ Mitchell Cancer Institute University of South Alabama Mobile Alabama USA; ^3^ Institute of Bioinformatics and Systems Biology, Helmholtz Zentrum München Neuherberg Germany; ^4^ German Center for Diabetes Research Neuherberg Germany; ^5^ Institute of Epidemiology II, Helmholtz Zentrum München Neuherberg Germany; ^6^ Metabolon, Inc Durham North Carolina USA; ^7^ Department of Statistics and Quantitative Methods University of Milano‐Bicocca Milan Italy; ^8^ Division of Cancer Epidemiology and Genetics National Cancer Institute, National Institutes of Health Bethesda Maryland USA; ^9^ School of Medicine, University of Nottingham Nottingham UK; ^10^ National Institute for Health Research, Nottingham Biomedical Research Centre Nottingham UK

## Abstract

**Objective:**

To investigate the association between long‐term weight change and blood metabolites.

**Methods:**

Change in BMI over 8.6 ± 3.79 years was assessed in 3,176 females from the TwinsUK cohort (age range: 18.3‐79.6, baseline BMI: 25.11 ± 4.35) measured for 280 metabolites at follow‐up. Statistically significant metabolites (adjusting for covariates) were included in a multivariable least absolute shrinkage and selection operator (LASSO) model. Findings were replicated in the Cooperative Health Research in the Region of Augsburg (KORA) study (*n* = 1,760; age range: 25‐70, baseline BMI: 27.72 ± 4.53). The study examined whether the metabolites identified could prospectively predict weight change in KORA and in the Prostate, Lung, Colorectal, and Ovarian Cancer (PLCO) study (*n* = 471; age range: 55‐74, baseline BMI: 27.24 ± 5.37).

**Results:**

Thirty metabolites were significantly associated with change in BMI per year in TwinsUK using Bonferroni correction. Four were independently associated with weight change in the multivariable LASSO model and replicated in KORA: namely, urate (meta‐analysis β [95% CI] = 0.05 [0.040 to 0.063]; *P* = 1.37 × 10^−19^), gamma‐glutamyl valine (β [95% CI] = 0.06 [0.046 to 0.070]; *P* = 1.23 × 10^−20^), butyrylcarnitine (β [95% CI] = 0.04 [0.028 to 0.051]; *P* = 6.72 × 10^−12^), and 3‐phenylpropionate (β [95% CI] = −0.03 [−0.041 to −0.019]; *P* = 9.8 × 10^−8^), all involved in oxidative stress. Higher levels of urate at baseline were associated with weight gain in KORA and PLCO.

**Conclusions:**

Metabolites linked to higher oxidative stress are associated with increased long‐term weight gain.

## Introduction

Obesity is a major public health concern resulting from a positive energy balance for an extended period of time 1. Although most people in industrialized nations have overweight or obesity, significant numbers of people with normal weight never develop overweight or obesity, partly reflecting the large interindividual variation in response to excess caloric intake 1. A clear understanding of the mechanisms of obesity and development of effective strategies for its prevention and treatment require consideration of biochemical changes resulting from weight gain and loss of metabolic homeostasis 2.

Comprehensive metabolic profiling provides an opportunity to gain new insights into biochemical differences involved in weight loss or gain in humans. Several metabolomic studies have been carried out to date, although most of them have focused on the overlap between obesity and type 2 diabetes mellitus 3. Dozens of metabolites fall into that category, including branched‐chain amino acids (BCAAs), cysteine, glutamine, phenylalanine, proline, tyrosine, threonine, tryptophan, choline, and pantothenic acid, which are increased in both obesity and diabetes. In contrast, asparagine, glycine, methionine, and citrulline are decreased in both obesity and diabetes 4. Metabolomic profiling of BMI and obesity independent of the link with diabetes has also been performed. In a cross‐sectional study of 947 participants, 37 metabolites were significantly associated with BMI, including 19 lipids, 12 amino acids, and 6 others. Eighteen of these associated metabolites had not been previously reported on, including histidine and butyrylcarnitine, a lipid marker of whole‐body fatty acid oxidation 5.

Similar results have been derived from smaller studies focused on child obesity. A study profiling serum samples of 80 children with obesity and 40 children with normal weight identified 14 metabolites (glutamine, methionine, proline, 9 phospholipids, and 2 acylcarnitines) that were significantly different when comparing children with normal weight and children with obesity 6.

Less research has been conducted to investigate the metabolites related not to high or low BMI but to change in BMI per year. One study investigated the metabolic adaptations seen after bariatric surgery. The metabolic footprint of bariatric procedures appears to be specifically characterized by an increase of bile acid circulating pools and a decrease of ceramide levels, a greater perioperative decline in BCAA, and the rise of circulating serine and glycine, mirroring glycemic control and inflammation improvement 7.

A recent study from the Cooperative Health Research in the Region of Augsburg (KORA) cohort defined metabolite modules or clusters of related molecules (using weighted correlation network) and then identified four metabolite modules significantly and stably associated with body weight gain. These included major branches of metabolism, with very low‐density lipoprotein, low‐density lipoprotein (LDL), and large high‐density lipoprotein subclasses, triglycerides, BCAAs, and markers of energy metabolism, among others 8.

However, a key question is “What are the molecular pathways that are associated with greater or lower weight change over time?” This is relevant if these factors are to be addressed and eventually modified for therapeutic purposes.

In this study, we used longitudinal measures of BMI in 3,176 women from the TwinsUK cohort assessed 8.6 years apart. Having adjusted for age and BMI at baseline, smoking, and family relatedness, we carried out metabolomic profiling of change in BMI per year. We replicated our results in 1,760 individuals from KORA and in an elderly cohort from the United States. Having identified metabolites whose levels are significantly and reproducibly altered by weight change, we then tested in a subanalysis whether the levels of any of these at baseline (before weight change) were also associated with weight change.

## Methods

### Discovery cohort

Study subjects were twins enrolled in the TwinsUK registry, a national register of adult twins recruited as volunteers without selecting for any particular disease or trait 9. All recruited twins were of the same sex. In this study, we analyzed data from 3,176 females (age range: 18.3‐79.6) from the TwinsUK cohort with BMI assessed on average 8.6 ± 3.79 years apart and metabolomic profiling conducted at follow‐up.

The study was approved by St Thomas' Hospital Research Ethics Committee, and all twins provided informed written consent.

### Replication cohorts

The replication cohort consisted of individuals from the follow‐up study (KORA F4) drawn from the general population of the region of Augsburg, Germany 10. The average follow‐up time from the baseline study (KORA S4) was 7.11 ± 0.22 years. In total, we analyzed 1,760 individuals (age range: 25‐70) with fasting serum metabolomic profiles made available using the Metabolon, Inc. (Durham, North Carolina) platform and with the measure of longitudinal BMI.

We tested whether the identified and replicated metabolites could also predict weight change on a different set of fasting and nonfasting samples from the KORA S4 study, which comprised 1,069 subjects (age range: 32‐77) with nontargeted metabolomic data at baseline and longitudinal BMI.

We also included 471 female controls (age range: 55‐74) from the Prostate, Lung, Colorectal, and Ovarian Cancer (PLCO) study 11. Eligibility criteria were that participants had to be controls, had to have a follow‐up weight measurement, and had to have metabolomic profiling available. For PLCO, metabolomic profiling was available only at baseline.

The PLCO trial is registered at ClinicalTrials.gov as NCT00002540, and it was approved by the institutional review boards of the US National Cancer Institute and the 10 screening centers. Only controls from this trial are included in this study. The KORA F4/S4 study was approved by the ethics committee of the Bavarian Medical Association.

### Assessment of weight gain, weight loss, and covariates

Height and weight were measured once at baseline and once at follow‐up, which was on average 8.6 ± 3.79 years after the baseline assessment. BMI was calculated by dividing weight (in kilograms) by the square of height (in meters).

For the subanalysis and for computing box plots of the metabolites' distribution, the change in BMI per year was calculated by adjusting for age, BMI at baseline, smoking, metabolite batch, and family relatedness. Subjects were then categorized based on these tertiles. The weight gain group was defined as the top tertile, while the low weight gain (weight loss) group was defined as the lower tertile 12. The middle tertile was excluded from the subanalysis.

### Metabolomics measurements

Nontargeted metabolite detection and quantification were conducted by the metabolomics provider Metabolon on 3,176 fasting samples from participants in the TwinsUK study, as described previously 13 (for details, see the Supporting Information). The metabolomic data set measured by Metabolon includes 280 metabolites of known chemical identity containing the following broad categories: amino acids, acylcarnitines, sphingomyelins, glycerophospholipids, carbohydrates, vitamins, lipids, nucleotides, peptides, xenobiotics, and steroids.

Circulating levels of saturated fatty acids (SFAs), total fatty acids (TotFAs), monounsaturated fatty acids, and polyunsaturated fatty acids (PUFAs) were measured by Nightingale Health, previously known as Brainshake Ltd. (Vantaa, Finland; https://nightingalehealth.com/services), from fasting serum samples using proton nuclear magnetic resonance spectroscopy at 500 MHz and 600 MHz, as previously described 14. Traits were natural log‐transformed and then scaled to standard deviation (SD) units, as previously proposed by Würtz et al 15. For the metabolites containing zeros, one was added to all values of that metabolite before natural log‐transformation.

### Statistical analysis

Statistical analysis was carried out using the data analysis and statistical software Stata version 11 (StataCorp LLC, College Station, Texas) and R version 3.3.1 (R Foundation for Statistical Computing, Vienna, Austria). We inverse normalized the metabolite data, as the metabolite concentrations were not normally distributed. We excluded metabolic traits with more than 20% of values missing. We imputed individual metabolites not detected in a sample using the minimum run‐day measures.

Linear regression analysis adjusting for age, BMI at baseline, smoking, metabolite batch, and family relatedness was undertaken to identify associations between biomarkers and weight change. We corrected for multiple testing using Bonferroni correction (*P* < 1.2 × 10^−4^). To identify a set of metabolites independently associated with weight change, we fitted the least absolute shrinkage and selection operator method (LASSO), incorporating all significant metabolites together with age, smoking, and metabolite batch. LASSO is a regression method that performs both variable selection and regularization. It involves penalizing the absolute size of the regression coefficients with some parameter estimates shrunk to zero 16. The penalty parameter was chosen as the largest λ such that error is within one standard error of the minimum as determined by cross validation using the glmnet package in R 17.

### Replication analysis

The metabolites independently associated with weight change were replicated in 1,760 individuals from the KORA cohort. Results were then combined using an inverse variance fixed‐effect meta‐analysis. The fixed‐effect model provides a weighted average of the study estimates, the weights being the inverse of the variance of the study estimate.

We computed the area under the curve (AUC) and 95% CIs for a receiver operating characteristic curve comparing the top and bottom tertiles of BMI change per year in the TwinsUK cohort. Two models were fitted: 1 demographics and lifestyle, including age, baseline BMI, and smoking and 2 all variables in 1 plus the inverse normal concentrations of the four successfully replicated metabolites. This allowed us to evaluate the extent to which the significant metabolites contribute to the AUC in addition to the demographic and lifestyle variables. As metabolites may also be predictive of BMI change per year, we finally tested the association of weight change and metabolites at baseline in both KORA and PLCO. Metabolomic data at baseline in twins were not available.

## Results

The descriptive characteristics of the study participants are presented in Table 1. Thirty metabolites were associated with weight change after adjusting for covariates and multiple testing using Bonferroni correction. We investigated whether these associations were sensitive to the various adjustments and found that results were consistent (Supporting Information Tables S1‐S2).

**Table 1:** Descriptive characteristics of the study population

	TwinsUK	KORA	PLCO
**Females (%)**	100	51	100
**Age at baseline**	50.26 ± 10.24	53.72 ± 8.79	62.65 ± 5.12
**Age at follow‐up**	58.86 ± 10.54	60.82 ± 8.79	71.74 ± 5.89
**BMI at baseline**	25.11 ± 4.35	27.72 ± 4.53	27.24 ± 5.37
**BMI at follow‐up**	26.38 ± 4.76	28.17 ± 4.81	27.60 ± 5.71
**Current smoker (%)**	8.09	14.77	5.94

Values represent the mean (SD) unless otherwise specified.

We then used a LASSO model to analyze these 30 metabolites, identifying 7 that contributed independently to weight change (Table 2). Of those, 4 were successfully replicated in the KORA cohort and were therefore meta‐analyzed using inverse variance fixed‐effect meta‐analysis. Urate (meta‐analysis β [95% CI] = 0.05 [0.040 to 0.063]; *P* = 1.37 × 10^−19^), gamma‐glutamyl valine (β [95% CI] = 0.06 [0.046 to 0.070]; *P* = 1.23 × 10^−20^), and butyrylcarnitine (β [95% CI] = 0.04 [0.028 to 0.051]; *P* = 6.72 × 10^−12^) were positively associated with weight gain, while 3‐phenylpropionate (hydrocinnamate) (β [95% CI] = −0.03 [−0.041 to −0.019]; *P* = 9.8 × 10^−8^ ) was inversely associated with weight change. Supporting Information Figure S1 shows the box plot of the distribution of the four metabolite concentrations (in the inverse normal scale) for each of the three tertiles of change in BMI per year. Tertile 1 refers to individuals with the lowest BMI change per year, while tertile 3 refers to the highest BMI gain over time.

**Table 2:** List of metabolites associated with weight change in the LASSO model

			TwinsUK			KORA			Fixed‐effect meta‐analysis	
Super	Sub	Metabolite	β^a^	SE	*P*	β^a^	SE	*P*	β (95% CI)	*P*
**Amino acid**	Phenylalanine and tyrosine metabolism	3‐Phenylpropionate (hydrocinnamate)	−0.03	0.006	3.01 × 10^−6^	−0.03	0.007	3.33 × 10^−6^	−0.03 (−0.041 to −0.019)	9.8 × 10^−8^
**Cofactors and vitamins**	Hemoglobin and porphyrin metabolism	Bilirubin (Z,Z isomer)	−0.03	0.006	2.30 × 10^−7^	−0.09	0.007	1.91 × 10^−1^		
**Lipid**	Fatty acid metabolism (also BCAA metabolism)	Butyrylcarnitine	0.04	0.007	7.88 × 10^−9^	0.04	0.007	8.63 × 10^−9^	0.04 (0.028 to 0.051)	6.72 × 10^−12^
**Lipid**	Lysolipid	1‐Docosahexaenoyl‐GPC (22:6)	−0.04	0.007	5.19 × 10^−9^	−0.02	0.007	1.20 × 10^−2^		
**Nucleotide**	Purine and urate metabolism	Urate	0.05	0.007	3.04 × 10^−13^	0.06	0.008	2.27 × 10^−15^	0.05 (0.040 to 0.063)	1.37 × 10^−19^
**Peptide**	Gamma‐glutamyl	Gamma‐glutamylvaline	0.06	0.008	8.28 × 10^−17^	0.04	0.008	1.62 × 10^−8^	0.06 (0.046 to 0.070)	1.23 × 10^−20^
**Peptide**	Polypeptide	HWESASXX	0.04	0.008	2.86 × 10^−9^	−0.01	0.007	1.48 × 10^−1^		

β values (SEs) are estimated with linear regressions adjusting for age, BMI at baseline, smoking, batch, and familiar relatedness. Results are combined (if replicated in the KORA study) using fixed‐effect meta‐analysis.

a β represents the yearly change in BMI per 1 SD increase of metabolite concentration. Analyses in KORA are additionally adjusted for sex.

To quantify how much of the change in BMI per year was explained by these four metabolites, we computed the AUC in a receiver operating characteristic analysis. We found that the AUC obtained by adding these four metabolites was significantly greater than for the lifestyle and demographic variables alone. Demographics plus smoking gave an AUC of 0.563 (95% CI: 0.539‐0.587), whereas the addition of the metabolite data resulted in an AUC of 0.637 (95% CI: 0.614‐0.660) (Supporting Information Figure S2).

The full metabolome has been previously investigated for its association with intake of 71 food groups 18. (The full information can be searched online at http://www.twinsuk.ac.uk/dietmetab-data/.) Of the metabolites identified here as related to weight change, urate, gamma‐glutamyl valine, and butyrylcarnitine were not associated with any of the 71 food groups that we considered. On the other hand, 3‐phenylpropionate (hydrocinnamate), which was associated with negative BMI change, was associated with higher consumption of apples and pears (β [SE] = 0.02 [0.004]; *P* = 1.24 × 10^−8^) and lower consumption of fried fish (β [SE] = −0.17 [0.05]; *P* = 4.12 × 10^−11^) and savory pies (β [SE] = −0.23 [0.04]; *P* = 3.72 × 10^−10^) 18.

We further tested whether baseline levels of these metabolites were also predictive of weight change and found that circulating levels of urate at baseline were associated with higher weight gain at follow‐up in both KORA (β [SE] = 0.02 [0.009]; *P* = 0.04) and PLCO (β [SE] = 0.04 [0.020]; *P* = 0.008) and in the meta‐analysis (β [95% CI] = 0.023 [0.007‐0.039]; *P* = 0.004).

**Figure 1:**
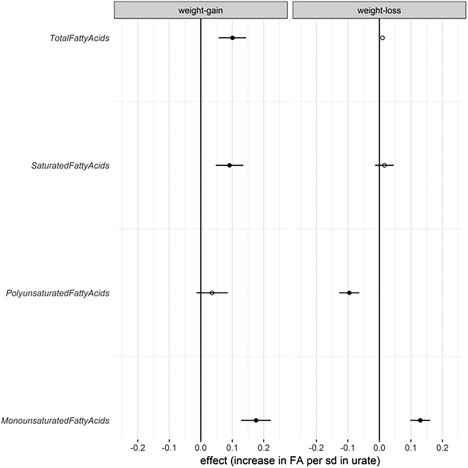
Association between circulating urate and fatty acids among participants with weight gain and those with weight loss. Linear regression coefficient β values (95% CIs) are depicted.

We hypothesized that urate may be a surrogate marker of the metabolic change or changes that result in weight gain. If this were true, we would expect levels of urate at baseline to be positively correlated with levels of fatty acids in blood in individuals who gain the most weight. If that were the case, there should be no such metabolic change in people who lose weight. Given the link to oxidative stress, we further hypothesized that this might affect SFAs and PUFAs differently. We therefore tested the interactions between urate and circulating levels of SFA, TotFA, monounsaturated fatty acid, and PUFA in the weight gain and weight loss groups (top and bottom tertiles of the weight change distribution, which had changes of −0.17 ± 0.26 and 0.39 ± 0.22 kg/m^2^ per year, respectively) and found that the association between urate and both SFAs and TotFAs was only present in those who gained weight (SFA: β [SE] = 0.09 [0.04]; *P* = 0.04; TotFA: β [SE] = 0.1 [0.04]; *P* = 0.02), while lower PUFAs were associated with higher circulating urate levels in those who lost weight (PUFA: β [SE] = −0.1 [0.03]; *P* = 0.002) (Figure 1).

## Discussion

In this study, we profiled the metabolic signatures of long‐term weight change. Having assessed the association between circulating metabolites and weight change, we identified four metabolites—urate, butyrylcarnitine, gamma‐glutamylvaline and 3‐phenylpropionate (hydrocinnamate), all of which have been previously linked to higher oxidative stress—that were independently associated with long‐term weight gain and successfully replicated in an independent cohort.

Previous studies reported that several metabolites—including BCAAs, butyrylcarnitine cysteine, glutamine, phenylalanine, proline, tyrosine, histidine, threonine, tryptophan, choline, pantothenic acid, LDL, very low‐density lipoprotein, asparagine, glycine, methionine, and citrulline, among others—were associated with obesity 3, 4, 5. Some of the metabolites we identified as related to weight change have been previously linked to obesity, specifically butyrylcarnitine, which was reported by Moore et al. in individuals of both Asian and European descent 5. We did not find a correlation with BCAAs, which are well‐known markers of visceral fat 19, obesity in general 7, and type 2 diabetes 3. Also, unlike the authors of previous studies 8, we did not find a strong correlation between weight change and lipids such as LDL or other forms of cholesterol. These findings are in line with those of studies of high fat and high sucrose intake with regards to the generation of reactive oxygen species (ROS). Treatment with high glucose or high fatty acid concentrations in cultured skeletal muscle cells has been shown to induce ROS production and mitochondrial damage in myotubes 20. In both rodents and humans, a high‐fat diet increased the hydrogen peroxide‐emitting potential of mitochondria without any changes in oxidative capacity 21. Not only does high‐fat, high‐sucrose feeding in mice result in increased ROS production, but this ROS production also results in mitochondrial dysfunction 20, 21. In light of this finding, a possible mechanism linking the metabolites identified in our study and previous research could be the strong link between a dysfunction in energy metabolism and ROS generation.

### Butyrylcarnitine

Butyrylcarnitine is an acylcarnitine, a type of compound derived from mitochondrial acyl‐CoA metabolism, and it has been associated with diet‐induced insulin resistance 22. Acylcarnitines comprise an acyl group esterified to L‐carnitine, which enables them to cross the mitochondrial membrane. Several recent studies show increased levels of multiple acylcarnitines in subjects with obesity and insulin resistance 23. Acylcarnitines are generated during incomplete fatty acid beta‐oxidation, and treatment of cultured murine and human myotubes with these compounds induces insulin resistance and oxidative stress 24. High levels of butyrylcarnitine are therefore indicative of incomplete beta‐oxidation and are likely causative of increased ROS.

### Gamma‐glutamylvaline

Gamma‐glutamylvaline, like other gamma‐glutamylated amino acids, is an established marker of oxidative stress and reflective of glutathione (GSH) turnover 25. GSH is the major redox buffer in the cell 26. It protects cellular components from oxidative damage of ROS such as hydrogen peroxide and organic peroxides via GSH peroxidases and also detoxifies the electrophilic metabolites of toxins 27. The intracellular redox environment can be measured through the redox state of the GSH disulfide/GSH couple 28. Under conditions of oxidative stress, intracellular GSH turnover is increased, activating the enzyme γ‐glutamyl transpeptidase to cleave extracellular GSH to release gamma‐glutamylated amino acids, which are taken up for intracellular GSH reconstitution. A drop in such amino acids has been reported in response to vitamin E treatment 25.

### 3‐phenylpropionate (hydrocinnamate)

Lower levels of the metabolite 3‐phenylpropionate (hydrocinnamate) were also reproducibly associated with weight gain. Hydrocinnamate is a phenolic compound 29 with well‐characterized antioxidant activity 30. Moreover, derivatives of this compound are known to have agonistic effects on peroxisome proliferator‐activated receptors with antidiabetic and lipid‐lowering activity 31.

### Urate

In humans, the final compound of purine catabolism is uric acid 32. Urate is a well‐known marker of intracellular prooxidant and inflammatory status. Although it has antioxidant activity extracellularly, it has been widely linked to increased risk of metabolic syndrome 33. We found urate to be associated with weight change in all cohorts (Table 2), with urate levels at baseline being correlated with higher weight gain at follow‐up.

Urate, despite being a major antioxidant in the human plasma, both correlates and predicts development of obesity, hypertension, and cardiovascular disease, conditions associated with oxidative stress 34. Urate is a prooxidant when forming radicals in reactions with other oxidants, and these radicals predominantly target lipids (LDL and membranes) rather than other cellular components. However, the hydrophobic environment created by lipids is unfavorable for the antioxidant effects of uric acid, and oxidized lipids thus convert uric acid into an oxidant 34. There is a substantial body of evidence indicating that uric acid has a direct effect on adipose tissue, and this effect has a redox‐dependent component 34, 35, 36. Moreover, hyperuricemia was previously found to be a mediator linking obesity with kidney disease and might, thus, increase oxidative stress due to decreasing renal function 37.

### Proposed biological mechanism

The metabolic signatures we saw therefore could be interpreted as follows: The increase in fatty acids and carbohydrates produces increased ROS (as witnessed by the accumulation of gamma glutamyl amino acids) and incomplete beta‐oxidation (accumulation of acylcarnitines), which in turn causes mitochondrial dysfunction linked to a dysregulation of the tricarboxylic acid cycle. This would result in decreased adenosine triphosphate production, and eventually mitochondrial DNA degradation, leading to the release of nucleotides, nucleosides, and nucleobases, which are metabolized to urate by oxidation.

In a subanalysis of TwinsUK cohort data we found that urate is correlated with increased levels of SFAs and TotFAs, but only in individuals who gain weight (Figure 1). On the other hand, PUFAs, which have an antioxidant effect themselves 38, are correlated with lower levels of urate, but only in individuals who lose weight. Our data therefore suggest that increases (even at normal levels) of urate may potentially be markers of the start of metabolic changes that result in long‐term weight gain. It also suggests that the metabolites identified in our study might be used to monitor the efficacy of therapies aimed at restoring mitochondrial function, such as ongoing clinical trials using nicotinamide adenine dinucleotide precursors 39. Urate is not only an easily measured marker that can be monitored in therapies aimed at reducing weight gain, but, importantly, may also be modified through the use of urate‐lowering therapy. We note, however, that this is merely one interpretation of the data, and we were unable to prove causality; therefore, other interpretations are possible.

The current study has several strengths. It used a nontargeted metabolomics approach that identified a wide range of biochemicals in addition to lipids. TwinsUK is a very large and extensively phenotyped cohort, and we replicated our results in two independent cohorts.

We also note several study limitations. Our discovery sample consisted of females only. Longitudinal metabolomic data were not available in all of the three cohorts. Also, we did not have a direct measure of oxidative phosphorylation and redox function in these subjects. The lack of metabolomic data at baseline in the discovery cohort did not allow us to test the predictive value of these metabolites on weight loss or gain.

Another important limitation is the possibility of confounding with total energy intake and expenditure. Adjustment using self‐reported measures from questionnaires on diet and physical activity did not make a difference to our findings, but these self‐report values are not objectively measured, are subject to bias, and have wide margins of error 40, 41. In this study, we were unable to adjust for objective measures of energy intake and expenditure, and therefore, the effects that we reported may be affected if adjusted by such measures. On the other hand, the four metabolites that we focused on were replicated in two independent studies, lending further confidence to the robustness of the four metabolomic associations reported here.

In conclusion, our study shows that metabolites linked to higher oxidative stress define a metabolic profile correlated with long‐term weight gain and that urate at baseline is associated with weight gain over time.

## Supporting information

Supporting Information Figure 1.

Supporting Information Figure 2.

Supporting Information Tables.

Supporting Information
