# Supplementary figures and images for: Metabolomic Profiling of Long‐Term Weight Change: Role of Oxidative Stress and Urate Levels in Weight Gain
Source: Obesity (Silver Spring). 2017 Jul 31;25(9):1618–24. doi: 10.1002/oby.21922 (PMC5601206; doi:10.1002/oby.21922)

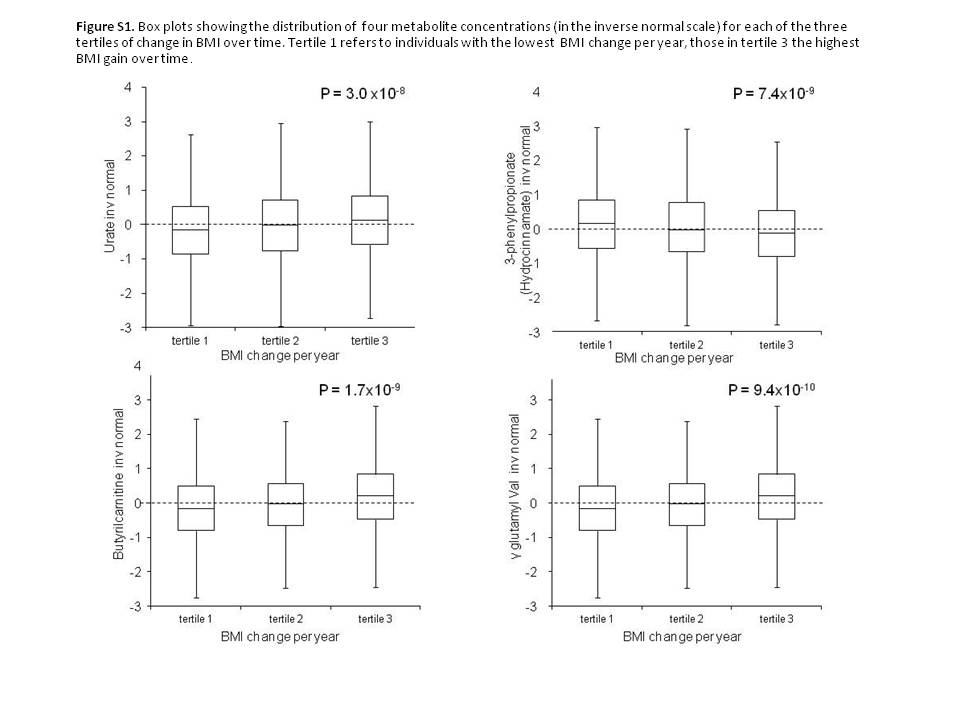

Supplement: Supplementary file 1 — Supporting Information Figure 1. [file OBY-25-1618-s001.jpg]

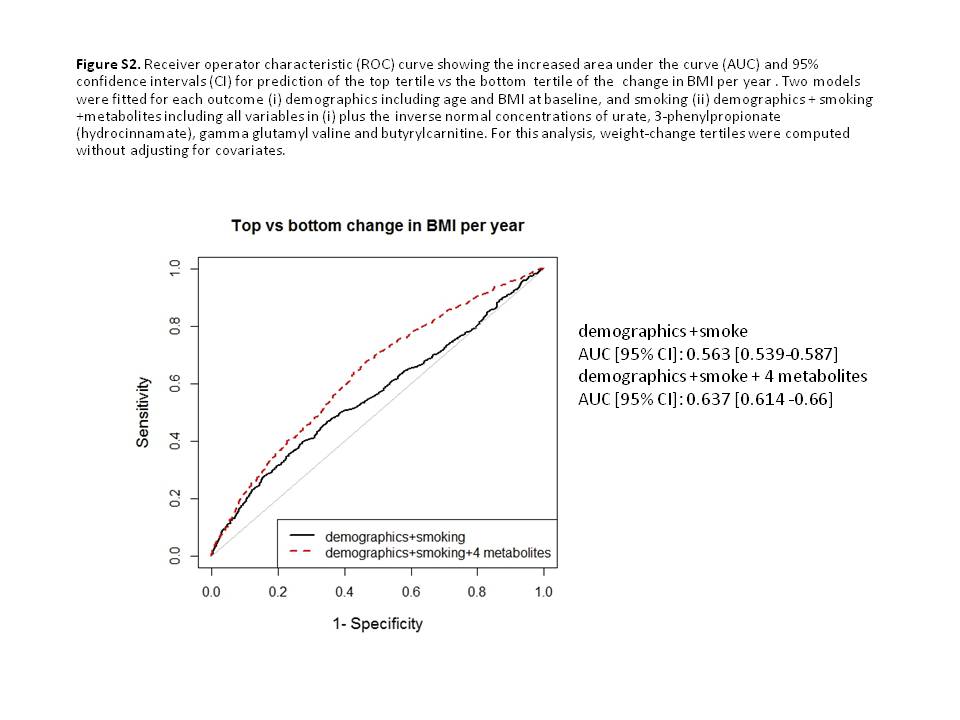

Supplement: Supplementary file 2 — Supporting Information Figure 2. [file OBY-25-1618-s002.jpg]
