# Supplementary material for: Metabolomic Profiling of Long‐Term Weight Change: Role of Oxidative Stress and Urate Levels in Weight Gain
Source: Obesity (Silver Spring). 2017 Jul 31;25(9):1618–24. doi: 10.1002/oby.21922 (PMC5601206; doi:10.1002/oby.21922)
Supplement: Supplementary file 3 — Supporting Information Tables. [file OBY-25-1618-s003.docx]

**Table S1. BMI per year and baseline non-metabolic variables**

|  | Beta(SE) | P |
| --- | --- | --- |
| BMI baseline | -0.005(0.002) | 0.03 |
| Age baseline | -0.003(0 .001) | 0.0001 |
| Smoking | 0.06(0 .03) | 0.02 |

**Table S2. List of metabolites associated with weight change in the TwinsUK cohort adjusting for age, BMI at baseline, smoking, metabolite batch, familiar relatedness and multiple testing using Bonferroni correction (P<1.2x10^-4^). Beta(SE) are also presented adding self reported energy intake intake physical activity and "follow-up duration" as covariates (columns 4-6) and for the unadjusted analysis (columns 7-10)**

|  | ***Analysis adjusted for age, BMI at baseline, smoking, metabolite batch and familiar relatedness*** | | | ***Analysis adjusted for age, BMI at baseline, smoking, calorie intake physical activity, metabolite batch, familiar relatedness and follow-up duration*** | | | ***Adjusting for metabolite batch and family relatedness*** | | |
| --- | --- | --- | --- | --- | --- | --- | --- | --- | --- |
| **Metabolite** | **Beta** | **SE** | **P** | **Beta** | **SE** | **P** | **Beta** | **SE** | **P** |
| N-acetylalanine | 0.032 | 0.007 | 6.51E-06 | 0.023 | 0.007 | 8.49E-04 | 0.031 | 0.007 | 1.23E-05 |
| glutamate | 0.044 | 0.007 | 2.47E-09 | 0.030 | 0.007 | 2.28E-05 | 0.044 | 0.007 | 4.91E-09 |
| lysine | 0.033 | 0.007 | 1.84E-06 | 0.027 | 0.007 | 6.26E-05 | 0.032 | 0.007 | 1.80E-06 |
| 3-phenylpropionate (hydrocinnamate) | -0.030 | 0.007 | 3.01E-06 | -0.028 | 0.006 | 1.37E-05 | -0.029 | 0.006 | 4.83E-06 |
| phenylalanine | 0.037 | 0.006 | 3.30E-11 | 0.031 | 0.005 | 1.31E-08 | 0.036 | 0.005 | 6.56E-11 |
| tyrosine | 0.037 | 0.006 | 1.17E-08 | 0.028 | 0.006 | 1.88E-05 | 0.036 | 0.006 | 2.13E-08 |
| C-glycosyltryptophan* | 0.043 | 0.006 | 2.14E-09 | 0.024 | 0.007 | 2.73E-04 | 0.041 | 0.007 | 1.81E-08 |
| kynurenine | 0.045 | 0.007 | 5.22E-12 | 0.032 | 0.006 | 1.09E-07 | 0.044 | 0.006 | 1.05E-11 |
| proline | 0.029 | 0.006 | 3.59E-07 | 0.026 | 0.006 | 1.03E-05 | 0.029 | 0.006 | 9.91E-07 |
| 2-methylbutyroylcarnitine | 0.040 | 0.006 | 4.78E-10 | 0.033 | 0.006 | 3.00E-07 | 0.042 | 0.007 | 7.88E-10 |
| isoleucine | 0.048 | 0.007 | 8.48E-13 | 0.041 | 0.007 | 3.91E-10 | 0.047 | 0.007 | 1.57E-12 |
| isovalerylcarnitine | 0.035 | 0.007 | 5.07E-08 | 0.028 | 0.006 | 6.80E-06 | 0.034 | 0.006 | 8.99E-08 |
| leucine | 0.042 | 0.006 | 1.11E-10 | 0.036 | 0.006 | 2.69E-08 | 0.041 | 0.006 | 1.57E-10 |
| valine | 0.049 | 0.006 | 2.32E-13 | 0.042 | 0.007 | 3.69E-10 | 0.049 | 0.007 | 2.85E-13 |
| mannose | 0.037 | 0.007 | 7.61E-06 | 0.027 | 0.008 | 1.21E-03 | 0.037 | 0.008 | 9.80E-06 |
| 1,5-anhydroglucitol (1,5-AG) | 0.027 | 0.008 | 5.30E-06 | 0.029 | 0.006 | 1.51E-06 | 0.027 | 0.006 | 7.19E-06 |
| bilirubin (Z,Z) | -0.033 | 0.006 | 2.30E-07 | -0.031 | 0.006 | 8.93E-07 | -0.032 | 0.006 | 2.98E-07 |
| succinylcarnitine | 0.038 | 0.006 | 9.60E-08 | 0.026 | 0.007 | 1.10E-04 | 0.037 | 0.007 | 3.47E-07 |
| butyrylcarnitine | 0.040 | 0.007 | 7.88E-09 | 0.033 | 0.007 | 2.45E-06 | 0.040 | 0.007 | 2.66E-08 |
| propionylcarnitine | 0.047 | 0.007 | 6.44E-13 | 0.035 | 0.006 | 1.71E-08 | 0.046 | 0.006 | 2.24E-12 |
| glycerol | 0.040 | 0.006 | 8.62E-06 | 0.029 | 0.009 | 1.07E-03 | 0.039 | 0.009 | 1.27E-05 |
| 1-docosahexaenoyl-GPC (22:6)* | -0.039 | 0.009 | 5.19E-09 | -0.041 | 0.006 | 4.42E-10 | -0.039 | 0.007 | 5.94E-09 |
| N1-methyladenosine | 0.025 | 0.007 | 1.01E-05 | 0.020 | 0.006 | 2.95E-04 | 0.024 | 0.006 | 2.60E-05 |
| urate | 0.049 | 0.006 | 3.04E-13 | 0.035 | 0.006 | 5.78E-08 | 0.048 | 0.007 | 7.82E-13 |
| gamma-glutamylleucine | 0.057 | 0.007 | 5.09E-15 | 0.043 | 0.007 | 8.87E-10 | 0.056 | 0.007 | 1.83E-14 |
| gamma-glutamylphenylalanine | 0.043 | 0.007 | 1.25E-11 | 0.031 | 0.006 | 3.72E-07 | 0.042 | 0.006 | 4.76E-11 |
| gamma-glutamyltyrosine | 0.045 | 0.006 | 1.49E-10 | 0.029 | 0.007 | 1.12E-05 | 0.044 | 0.007 | 3.22E-10 |
| gamma-glutamylvaline | 0.063 | 0.007 | 8.28E-17 | 0.048 | 0.007 | 5.90E-11 | 0.063 | 0.008 | 3.01E-16 |
| HWESASXX* | 0.045 | 0.008 | 2.86E-09 | 0.041 | 0.007 | 4.88E-08 | 0.044 | 0.008 | 6.23E-09 |
|  |  |  |  |  |  |  |  |  |  |

***** Self reported caloric intake was estiamted from food frequency questionnaire (FFQ) [^14^](#_ENREF_14). Macro and micro nutrient intakes were calculated from an established nutrient database[^15^](#_ENREF_15). A physical activity questionnaire derived from the older version of the Behavioral Risk Factor Surveillance System (BRFSS)[^17^](#_ENREF_17) was applied to assess leisure time, household, and transportation-related activity of moderate- and vigorous intensity
