# Supplementary material for: Metabolomic Profiling of Long‐Term Weight Change: Role of Oxidative Stress and Urate Levels in Weight Gain
Source: Obesity (Silver Spring). 2017 Jul 31;25(9):1618–24. doi: 10.1002/oby.21922 (PMC5601206; doi:10.1002/oby.21922)
Supplement: Supplementary file 4 — Supporting Information [file OBY-25-1618-s004.docx]

*Metabolomics measurements*

Proteins were precipitated and chemically diverse metabolites were isolated with methanol under vigorous shaking for 2 minutes followed by centrifugation. The resulting extract was divided into four fractions: one for analysis by ultra-high performance liquid chromatography-tandem mass spectrometry (UPLC-MS/MS; positive mode), one for analysis by UPLC-MS/MS (negative mode), one for analysis by gas chromatography–mass spectrometry (GC-MS), and one sample was reserved for backup. Three types of controls were analyzed in concert with the experimental samples: samples generated from a pool of human plasma (extensively characterized by Metabolon, Inc.) served as technical replicates throughout the data set; extracted water samples served as process blanks; and a cocktail of standards spiked into every analyzed sample allowed instrument performance monitoring. Experimental samples and controls were randomized across the platform run.

The UPLC-MS/MS platform utilized a Waters Acquity UPLC and a ThermoFisher LTQ mass spectrometer, which included an electrospray ionization source and a linear ion-trap mass analyzer. The instrument was set to scan 99-1000 m/z and alternated between MS and MS/MS scans. The instrumentation was set to monitor for positive ions in acidic extracts or negative ions in basic extracts through independent injections. Extracts were loaded onto columns (Waters UPLC BEH C18-2.1 × 100 mm, 1.7 μm) and gradient-eluted with water and 95% methanol containing 0.1% formic acid (acidic extracts) or 6.5 mM ammonium bicarbonate (basic extracts). Columns were washed and reconditioned after every injection.

Samples analyzed by GC-MS were dried under vacuum desiccation for a minimum of 18 h prior to being derivatized under dried nitrogen using bistrimethyl-silyl trifluoroacetamide. Derivatized samples were separated on a 5% phenyldimethyl silicone column with helium as carrier gas and a temperature ramp from 60° to 340° C within a 17-min period. All samples were analyzed on a Thermo-Finnigan Trace DSQ MS operated at unit mass resolving power with electron impact ionization and a 50-750 atomic mass unit scan range.

Metabolites were identified by automated comparison of the ion features in the experimental samples to a reference library of chemical standard entries that included retention time, molecular weight (m/z), preferred adducts, and in-source fragments as well as associated MS spectra and curated by visual inspection for quality control using software developed at Metabolon ([1](#_ENREF_1)). Identification of structurally named chemical entities is based on comparison to a mass spectroscopy library of >2,400 purified standards. An additional 5,300 mass spectral entries have been created for structurally unnamed biochemicals, which have been identified by virtue of their recurrent nature (both chromatographic and mass spectral). These compounds have the potential to be identified by future acquisition of a matching purified standard or by classical structural analysis. Peaks were quantified using area under the curve.

*Quality control of the metabolomics dataset*

Metabolomic profiling was done in three batches and on both serum and plasma samples.

We median-normalised the raw data by dividing each metabolite concentration by the day metabolite median, then inverse normalised the data as the metabolite concentrations were not normally distributed. Also, to avoid spurious false-positive associations due to small sample size, we excluded metabolic traits with more than 20% missing values.

We imputed the missing values using the minimum run day measures.

**Reference**:

1. Dehaven CD, Evans AM, Dai H, Lawton KA. Organization of GC/MS and LC/MS metabolomics data into chemical libraries. J. J Cheminform. 2010 Oct 18;2(1):9. doi: 10.1186/1758-2946-2-9.
